# Emergence of highly virulent and multidrug-resistant *Escherichia coli* in breeding sheep with pneumonia, Hainan Province, China

**DOI:** 10.3389/fmicb.2024.1479759

**Published:** 2024-10-23

**Authors:** Mengqi Wang, Xuesong Li, Guiying Guo, Muhammad Nafees Ur Rehman, Xiaomeng Gao, Lixia Fan, Nuo Yang, Jifeng Zeng, Jiping Zheng

**Affiliations:** ^1^Lab of Microbiological Engineering (Infection and Immunity), School of Life and Health Sciences, Hainan Province Key Laboratory of One Health, Collaborative Innovation Center of One Health, Hainan University, Haikou, China; ^2^Hainan International One Health Institute, Hainan University, Haikou, China; ^3^School of Chemical Engineering and Technology, Hainan University, Haikou, China

**Keywords:** *Escherichia coli*, pneumonia, multidrug-resistance, highly virulent, crosshost transmission, zoonotic potential

## Abstract

**Background:**

Sheep are a rarely raised livestock in Hainan Island, China, because of the unfavorable tropical marine climate. Here, this article reports a severe pneumonia in the sheep breeding and domestication facility caused acute mortality during the winter 2021–2022.

**Methods:**

Six sheep were clinically dissected and histopathologically observed. The bacteria were isolated and cultured by traditional methods and identified by 16S rRNA sequencing. The genotypes, serotypes, virulence genes and antimicrobial resistance genes were analyzed by PCR and whole genome sequencing. The pubMLST website was used for phylogenetic analysis of related strains. Kirby-Bauer disk diffusion method was used for antimicrobial susceptibility test. The antimicrobial susceptibility test standard was referred to the Clinical and Laboratory Standards Institute (CLSI). The virulence of bacteria was detected by mouse infection model.

**Results:**

Etiology and histopathology examination of the pneumonia reveled pulmonary abscess and alveolar neutrophilia and pulmonary fibrinous exudates. *Escherichia coli* was the only bacterial species isolated, primarily from the lungs and blood of the six dead or moribund sheep, a total of 29 *E. coli* strains were isolated. Antimicrobial resistance profiling shows that all the isolates were resistant to six agents (penicillin, ampicillin, cephalothin, neomycin, erythromycin, and vancomycin) belonging to five classes of antibiotics, classifying them as multi drug resistant (MDR). Furthermore, genotyping analysis revealed all strains were common with 11–17 virulence factors indicating high pathogenicity. The lab mice infection model shows that all strains severely affect the health status particularly weight loss, lethargy, pneumonia and shortly lead to death. The molecular epidemiological analysis indicated most strains share the same genotype as previously reported strains in humans and other farmed animals this suggests a high possibility of cross-species transmission (CST) of virulent and MDR isolates. This CST could be from sheep to humans and other farmed animals or from humans and other farmed animals to sheep.

**Conclusion:**

Therefore, this study indicates that *E. coli* is an emerging threat that causes sheep pneumonia in Hainan, and the quarantine of contacts is important to control the spread of virulent *E. coli* and the transmission of acquired resistance genes between humans and farmed animals such as sheep.

## Background

*Escherichia coli*, a gram negative, genetically diverse bacterium includes both beneficial strains colonizing the gut and highly pathogenic strains which is responsible for intestinal and extraintestinal diseases. The emergence of intestinal pathogenic *E. coli* (IPEC) and extraintestinal pathogenic *E. coli* (ExPEC) presents ongoing health concerns (Poolman and Wacker, 2016). These pathogenic strains cause a wide range of host infective syndromes, ranging from simple diarrhea and urinary tract infections (UTIs) to life-threatening septicemia or bacteremia. Furthermore, the appearance of multidrug-resistant (MDR) strains poses a significant challenge to the current treatments, fueling the global spread of antimicrobial resistance (Russo and Johnson, 2003; Mellata, 2013).

Certain epidemiological surveillance findings highlight the zoonotic risks posed by the highly virulent and MDR strains (Desvars-Larrive et al., 2024). These measures include using multi-locus sequence typing (MLST), pulsed field gel electrophoresis (PFGE), and virulence gene profiles (Maluta et al., 2014; Rodriguez-Siek et al., 2005) to compare the genetic relatedness between human and avian isolates acquired from poultry products, as well as between avian and human isolates from urine or blood sources. Previous studies have reported cross-species transmission from human to poultry and from poultry to mice (Tivendale et al., 2010; Zhao et al., 2009). Sheep, a widely distributed livestock species in the mainland China are rarely raised in the southernmost areas, such as Hainan Island due to the hot and humid climate (Gencay, 2014). To meet the demand of the growing tourist industry and provide for the nutritional needs of locals, sheep farming has been introduced to the area. However, this harsh and new habitat can adversely affect the farmed sheep, making them more susceptible to various infections including bacterial, viral and fungal diseases (Zhao J. Y. et al., 2021; Zhao X. et al., 2021; Kuraz Abebe, 2022). This can lead to a decline in livestock productivity and profitability, consequently damaging the economic health of the industry. Previous studies reported that the most common diseases among sheep are respiratory diseases such as pneumonia (Scott, 2011; Mekibib et al., 2019). Bacterial bronchopneumonia tends to occur most frequently in sheep that have experienced recent stressors, such as transportation, dietary changes, or mixing with herds from unrelated farms (Aiello and Moses, 2016). The most prevalent bacterial pathogens associated with the primary or secondary bronchopneumonia in sheep are including *Mannheimia haemolytica*, *Mycoplasma ovipneumoniae*, *Pasteurella multocida*, *Bibersteinia trehalosi*, *Chlamydia pneumoniae*, and *Salmonella* spp. (Besser et al., 2013; Alley et al., 1999). In rare cases, however, *E. coli* species have also been isolated from lung or heart blood samples of sheep with pneumonia and septicemia (Singh et al., 2019).

Here, we provide evidence that *E. coli* was the sole predisposing agent responsible for severe respiratory infections in a newly established sheep-breeding enterprise in Wenchang, Hainan (E110.78°, N19.96°), during winter 2021 to 2022. Additionally, the risks of cross-species transmission were investigated by ascertaining the antimicrobial resistance, virulence, and genetic characteristics of the *E. coli* isolates.

## Materials and methods

### Animal collection and gross examination

Sporadic deaths were reported in a newly established breeding farm in Wenchang, Hainan during the winter 2021–2022 following the introduction of new breeds. During this period, a total of six dead sheep including five adults and a lamb were collected for necropsy. According to a previous study (Rahsan et al., 2018), clinical signs including wounds, ulcers, skin lesions, excrements, and secretions from nose, eyes and mouth of each carcass were recorded before autopsy. Following dissection, bronchial exudates and lesions of the internal organs were examined visually and multiple systematic incisions.

### Histopathological examination

Following collection, tissue samples from lung, liver and other organs were fixed with 10% neutral buffered formalin. Subsequently, the samples were sent to Wuhan Saville Biotechnology Co., Ltd.^1^ for paraffin embedding, sectioning and staining. The stained slides were examined under a light microscope at 10× and 100× for histopathological evaluation (Rehman et al., 2021; Mekibib et al., 2019).

### Bacterial examination

For mycoplasma detection, a piece of lung tissue (0.2–0.5 g) was homogenized and centrifuged at 8, 000 rpm for 10 min followed by DNA extraction from 200 μL supernatant using the TIANamp Genomic DNA Kit (TIANGEN, Beijing, China). Specific primers were designed (Supplementary Table S1) and used in a polymerase chain reaction (PCR) (Young et al., 2010). In this paper, double-distilled water (ddH₂O) is employed as the negative control in all PCR. Furthermore, for bacterial isolation samples from various internal organs, blood, pleural effusions and bronchial exudates were inoculated onto brain heart infusion (BHI) agar plates following the method described by Rehman et al. (2021). Subsequently, the colonies were then primarily identified based on the conventional biochemical methods (Jia et al., 2022). For molecular identification, DNA was extracted from the samples using DNA isolation mini kit (Novazan, Nanjing, China) and 16S rRNA gene was amplified with the universal primers listed in Supplementary Table S1 followed by sequencing (Nanshan Biotech). Finally, the species level of each isolate was identified as matching the reference strain based on the new canonical clustering threshold of >99% similarity of full-length 16S rRNA gene (Edgar, 2018).

### Extraintestinal pathogenic *Escherichia coli* identification

*E. coli* isolates were tested by PCR with primer sets (Supplementary Table S1) of the following five virulence marker genes associated with ExPEC: *pap*A/C (P fimbriae; counted as 1), *sfa*/*foc* (S and F1C fimbriae), *afa*/*dra* (A fimbrial and Dr-binding adhesion), *kps*M II (group 2 capsule), and *iut*A (aerobactin system). Isolates positive for two or more of these markers were classified as ExPEC (Johnson et al., 2003).

### Phylogrouping and MLST analysis

The *E. coli* isolates were characterized according to phylogrouping and multilocus sequence typing (MLST) analysis. Phylogroups were determined using the Clermont PCR method (Clermont et al., 2013) (primers listed in Supplementary Table S1). In addition, MLST was performed using two schemes: the Achtman scheme targeting seven house-keeping genes (*adk, fumC, gyrB, icd, mdh, recA* and *purA*) (Manges et al., 2015), and the Pasteur scheme targetting eight housekeeping genes (*dinB, icdA, pabB, polB, putP, trpA, trpB* and *uidA*) (Primers in Supplementary Table S1) (Clermont et al., 2015).

### Serotype identification

Whole genome sequencing (WGS) was performed using Illumina HiSeq^™^ platform (PE150) at Sangon Bioengineering Co., Ltd. (Shanghai, China) on representative isolate of each *E. coli* sequence type. Serotypes were determined for these isolates using online SerotypeFinder2.0 services (Joensen et al., 2015). PCR based serotyping with primers designed for the representative isolates (Supplementary Table S1) was subsequently used to identify serotypes of the remaining isolates.

### Antimicrobial susceptibility assay

For antimicrobial susceptibility assay, Kirby-Bauer disc diffusion method was used for 19 drugs following the CLSI guidelines (Humphries et al., 2021). However, for two drugs polymyxin B and tigecycline (Solarbio Life Sciences Co, Beijing, China), the minimal inhibitory concentration (MIC) was determined according to the 2017 EUCAST methods.^2^ The antibiotics employed altogether fall into 11 major categories. Firstly, β-lactams include ampicillin, amoxicillin, and penicillin. Secondly, aminoglycosides consist of streptomycin, neomycin, and gentamicin. Thirdly, chloramphenicols are represented by chloramphenicol and florfenicol. Fourthly, fluoroquinolones comprise norfloxacin, ofloxacin, ciprofloxacin, and nalidixic acid. Fifthly, tetracyclines consist of tetracycline and tigecycline. Sixthly, cephalosporins are exemplified by cephalothin. Seventhly, sulfonamides include sulfisoxazole and trimethoprim. Eighthly, polypeptides are represented by polymyxin B. Ninthly, carbapenems are embodied by imipenem. Tenthly, macrolides are exemplified by erythromycin. Lastly, glycopeptides are represented by vancomycin. While *E. coli* ATCC 25,922 was subjected as quality control. Furthermore, the resistance gene pattern of each isolate was screened by PCR with the primer sets of 19 acquired antimicrobial resistance genes (Supplementary Table S1).

### Virulence gene profiling

Based on the WGS of each representative *E. coli* isolate, the virulence factors were further obtained by VFDB tool^3^ following the method of Rehman et al. (2023).

### Core genome multilocus phylogenetic analysis

Phylogenetic relationship between the isolated strains in this study and previously observed *E. coli* strains was determined according to a previous study with slight modifications (Rehman et al., 2023). The GrapeTree tool was used from pubMLST^4^ to analyze core genome multilocus phylogenetic analysis (cgMLPA) data for the isolated strains and other *E. coli* strains (Supplementary Table S2). iTOL v.4^5^ program was used for visualization and incorporation of available metadata into a phylogenetic tree.

### Experimental animal and ethics statement

Specific pathogen free (SPF) KM mice were employed from the Laboratory Animal Center, School of Pharmaceutical Sciences, Hainan University (SYXK0230021). The experimental protocol was approved by the Animal Management and Ethics Committee of Hainan University (HNUAUCC-2023-00020), and adhered to the recommendations of the Animal Ethics Procedures and Guidelines of the People’s Republic of China.

### Experimental design and infection model in mice

Mice were randomly allocated into six groups (five challenge groups and one control group) with six mice per group. The weight of each mouse was documented. Intraperitoneal injections were administered following the method of Long et al. (2022). Challenge groups were injected with 0.2 mL of *E. coli* solution (approximately 2 × 10^6^ CFU/mL), while control group received 0.2 mL PBS solution. Mice mortality rate and weight fluctuations were monitored every 6 h for 72 h. Pathological changes in the vital organs (heart, lung, liver, kidney and spleen) were recorded, and lungs were sent to Wuhan Saville Biotechnology Co., Ltd. (see text footnote 1) for further histopathological analysis.

## Results

### Necropsy and histopathological examination

**Figure 1:**
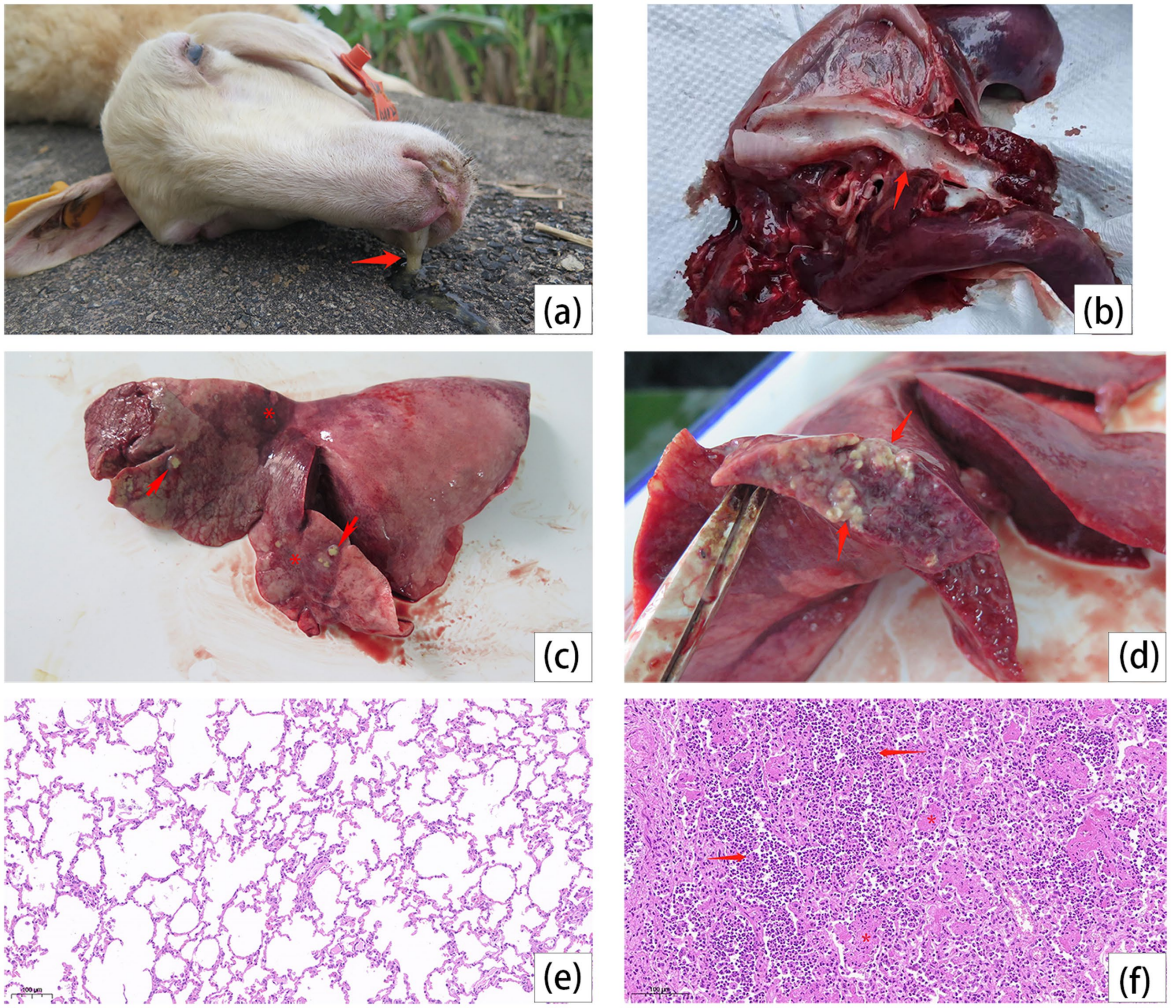
Dead sheep autopsy, lesions on trachea and lungs, and lung histopathology. **(a)** Secretions from the nasal cavity of the dead sheep (arrow). **(b)** White secretions in the trachea (arrow). **(c)** Lobar pneumonia in the left cranial and medial lobes. Irregular red consolidation in the affected lobe (stars), with varying sizes of abscesses (arrows). **(d)** The abscess infiltrates the lung (arrows). **(e)** The histopathological observation of lung in healthy sheep. **(f)** The alveoli are filled with dense neutrophils (arrows) and some alveoli have fibrin exudate in the lumen (stars).

Bronchopneumonia was diagnosed in six autopsied sheep collected at different time intervals. The common signs of the disease were sticky mucus in nostrils (Figure 1a) and trachea (Figure 1b), as well as irregular red consolidation and large-scale abscesses on the surfaces of extremely enlarged lungs (Figure 1c). These abscesses likely resulted from the internal accumulation of abundant pale-yellow mucoid exudate. (Figure 1d). Histopathological sectioning of lungs further revealed the features of lobular pneumonia of alveoli obliteration with the infiltration of neutrophils and capillary occlusion with intra-lumen fibrin exudates (Figure 1e,f).

### Bacterial identification

Gram-negative, rod-shape bacteria were isolated from various tissues, especially lungs, liver, and blood. Further, PCR detection with specific primers for *M. ovipneumoniae* and *M. mycoides* was negative. Based on traditional 16S rRNA sequencing, all the isolates were identified as *E. coli* (Table 1).

**Table 1:** *E. coli* isolated from different organs of six dead sheep.

Isolates	Host	Source	Collection time	Pathotype
Y2Q	Sheep1	Trachea	2021.10.21	IPEC
Y2G1	Sheep1	Liver	2021.10.21	IPEC
Y2G3	Sheep1	Liver	2021.10.21	IPEC
Y2P2	Sheep1	Spleen	2021.10.21	IPEC
Y2P3	Sheep1	Spleen	2021.10.21	IPEC
Y2Q3	Sheep1	trachea	2021.10.21	IPEC
Y2S1	Sheep1	Kidney	2021.10.21	IPEC
Y2S3	Sheep1	Kidney	2021.10.21	IPEC
Y2X3	Sheep1	Blood in the heart	2021.10.21	IPEC
Y2X4	Sheep1	Blood in the heart	2021.10.21	IPEC
Y2XS	Sheep1	Interstitial fluid in the chest	2021.10.21	IPEC
Y2XX1	Sheep1	Blood in the chest	2021.10.21	IPEC
Y2XX2	Sheep1	Blood in the chest	2021.10.21	IPEC
Y2F2	Sheep1	Lung	2021.10.21	IPEC
Y2P1	Sheep1	Spleen	2021.10.21	IPEC
Y2X1	Sheep1	Blood in the heart	2021.10.21	IPEC
Y3G1	Sheep2	Liver	2021.11.10	IPEC
Y3Q3	Sheep2	Trachea	2021.11.10	IPEC
Y3X1	Sheep2	Blood in the heart	2021.11.10	IPEC
Y3YF1	Sheep2	Right lung	2021.11.10	IPEC
Y3ZF5	Sheep2	Left lung	2021.11.10	IPEC
Y5P1	Sheep3	Spleen	2021.12.20	ExPEC
Y5YF3	Sheep3	Right lung	2021.12.20	ExPEC
Y5ZF1	Sheep3	Left lung	2021.12.20	ExPEC
Y11S1	Sheep4	Kidney	2022.04.08	IPEC
Y11S2	Sheep4	Kidney	2022.04.08	IPEC
Y12F1	Sheep5	Lung	2022.04.15	IPEC
Y12G1	Sheep5	Liver	2022.04.15	IPEC
Y13F1	Sheep6	Lung	2022.05.05	IPEC

### ExPEC identification

A total of 29 isolates were recovered and considered for ExPEC identification. In term of pathogenic *E. coli*, ExPEC strains are responsible for the majority of human or animal extraintestinal infections, including pneumonia, urinary tract infections, and sepsis. However, according to the classic ExPEC virulence genes defined by Johnson et al. (2003), our results demonstrated that only three are categorized in ExPEC (Table 1). Interestingly, all 29 isolates originated from outside of the gastrointestinal tract. This finding suggests that the current method may not effectively differential between facultative ExPEC pathogens and commensal *E. coli*.

### Phylogrouping and MLST analysis

**Figure 2:**
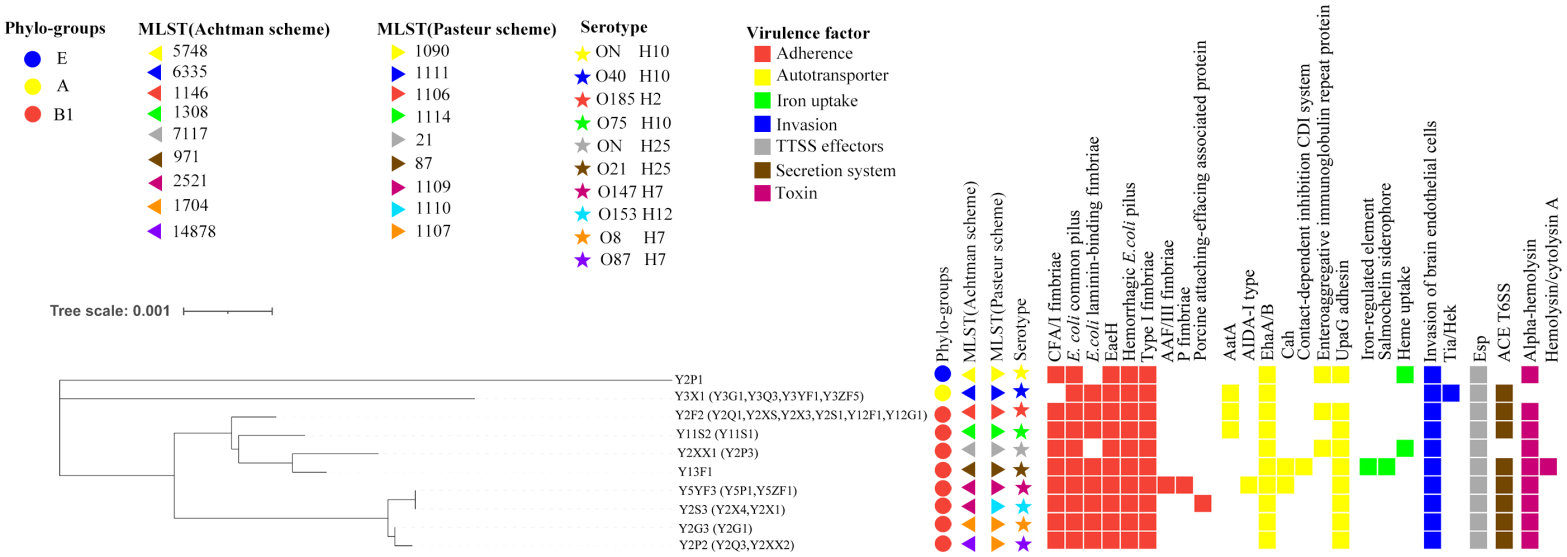
Phylo-groups, genotypes, serotypes, virulence factors of the 29 *E. coli* in this study.

A total of three phylogroups were predicted and among 29 isolates, five belonged to A, 23 belonged to B1, and only one belonged to E (Figure 2). According to the two MLST schemes, the isolates were further subdivided into nine sequence types (ST) (Figure 2). Three isolates (spleen, blood, and trachea) from the second sheep exhibited novel ST according to Achtman scheme. This new ST was closely related to ST1704, but it remained as unassigned as a new identifier. Interestingly, Pasteur scheme classified this same ST as the existing ST1107 (Figure 2). Moreover, most of the STs in this study were very consistent between the both MLST schemes, except ST2521 (Achtman), which was subdivided into ST1109 and ST1110 in the Pasteur MSLT scheme, and ST1107 of Pasteur scheme into ST1704 and ST14878 based on the Achtman scheme (Figure 2).

### Classification of serotype

WGS and PCR data were combined to classify the 29 isolates. According to the analysis, eight serotypes were obtained including both lipopolysaccharide O antigen and flagella H antigen. One exception case was the O-antigen serotype of three isolates (Y2XX1 from blood and Y2P1 and Y2P3 from sheep 2 spleen) was completely unmatched in the database of the Center for Genomic Epidemiology.^6^ This could be due to sequencing gaps within O-antigen and H-antigen gene clusters in their assembled genomes (Figure 2). By comparing the correlations of serotypes and ST, the serotypes O147:H7 and O153:H12 subdivided the isolates of ST2521 (Achtman scheme) into ST1109 and ST1110 (Pasteur scheme). Similarly, the O26:H11 and O8:H7 serotypes distinguished isolates ST1107 (Pasteur scheme) into ST1704 and ST14878 (Achtman scheme) (Figure 2). These finding suggest that the serotyping may be more effective in resolving the genetic distance between isolates than sequence typing. However, the two MLST schemes (Achtman and Pasteur) can be applied for co-analysis.

### Virulence factors detection

WGS data of 10 isolates representing unique serotypes were analyzed using the VFDB database. A total of 25 virulence factors categorized into seven classes were predicted. The most prevalent virulence factors included six adhesion-associated elements (CFA/I fimbriae, pilus, laminin-binding fimbriae, EaeH, hemorrhagic *E. coli* pilus, and type I fimbriae), two autotransporter associated elements (EhaA/B and UpaG adhesin), one invasion-associated element (invasion protein), two secretion system factors (T3SS translocator protein and Esp family protein), and one toxin factor (hemolysin/cytolysin A). All strains harbored at least 11 virulence factors. The highest and lowest virulence factors were predicted in isolates Y13F1 (17) and Y3X1 (11), respectively (Figure 2).

### Mouse infection model

**Figure 3:**
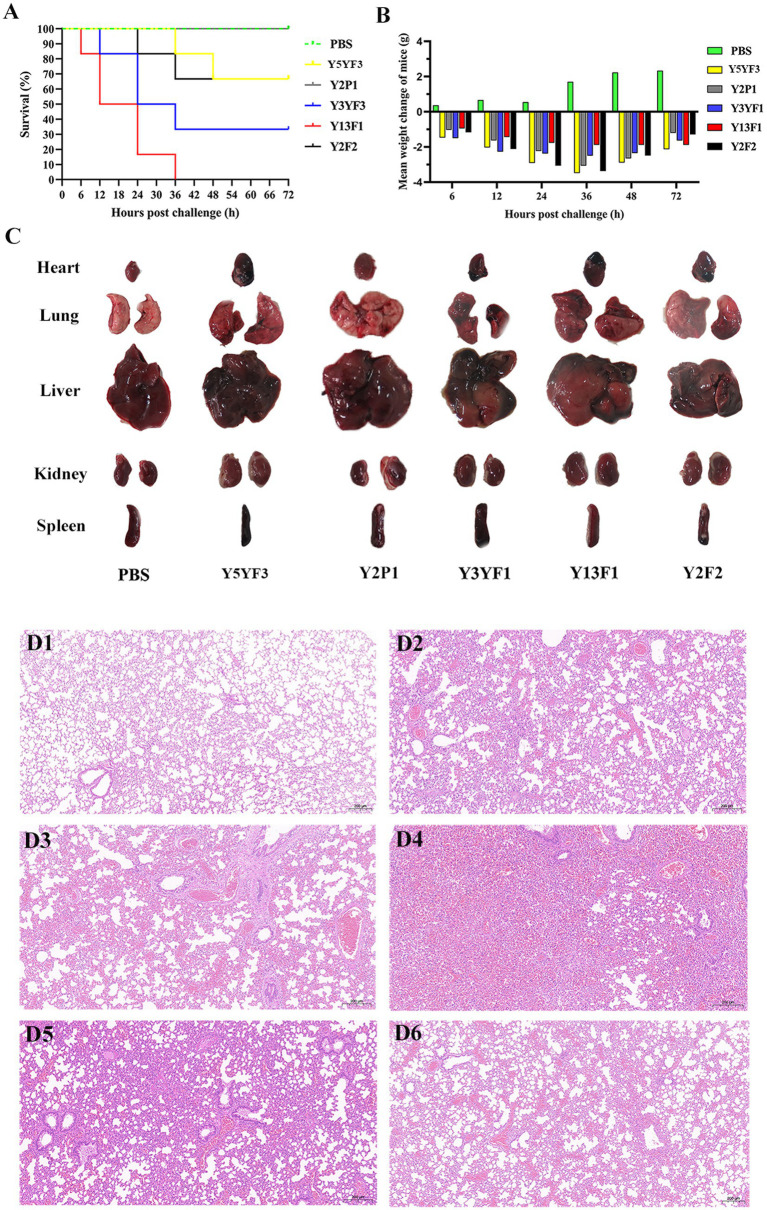
The results of pathogenicity test on mice. **(A)** Mouse survival rate curve. **(B)** Mean weight change of mice. **(C)** The main organs of the experimental mouse. **(D)** The histopathological observation of lung in healthy sheep (**D1–D6** separately represent the group of PBS, Y5YF3, Y2P1, Y3YF1, Y13F1, Y2F2).

To assess the pathogenicity of isolated strains with different virulence factor combinations, a mouse infection model was established. Mortality rate and weight changes were analyzed after intraperitoneal injection of isolates. The Y13F1 isolate exhibited the most lethal effect, resulting in the death of all mice within 36 h. Conversely, Y2P1 isolate demonstrated the least lethal effect and did not cause any mortality (Figure 3A). By examining the average weight changes of mice post-intraperitoneal injection, a significant increase in weight was noticed for the control group. All mice within the challenge group experienced weight loss, with Y5YF5 resulting in the most significant weight loss and Y13F1 the least (Figure 3B). Gross pathology examination shows that the main organs of all experimental mice exhibited either localized or extensive hemorrhages except control mice. Lung hemorrhages were most severe in mice challenged with Y13F1 and Y3YF1 isolates compared to other experimental groups (Figure 3C). Additionally, the spleens of Y2P1 and Y3YF1 challenged mice were notably swollen, while the spleens of Y5YF3 and Y3YF1 challenged mice appeared more blackened (Figure 3C).

Histopathological changes in the lungs show inflammatory responses compared to the control group. The lung inflammatory response in the Y3YF1 group was particularly intense, characterized by inflammatory cell infiltration, fibrinoid exudation, and bronchial epithelial cell shedding (Figure 3D). This study demonstrates that isolates with different virulence factor combinations exhibit variation in lethality, target organ tropism, and their capacity to cause pneumonia. However, all strains displayed a degree of pathogenicity, highlighting the importance of further investigation into these isolates.

### cgMLPA analysis

**Figure 4:**
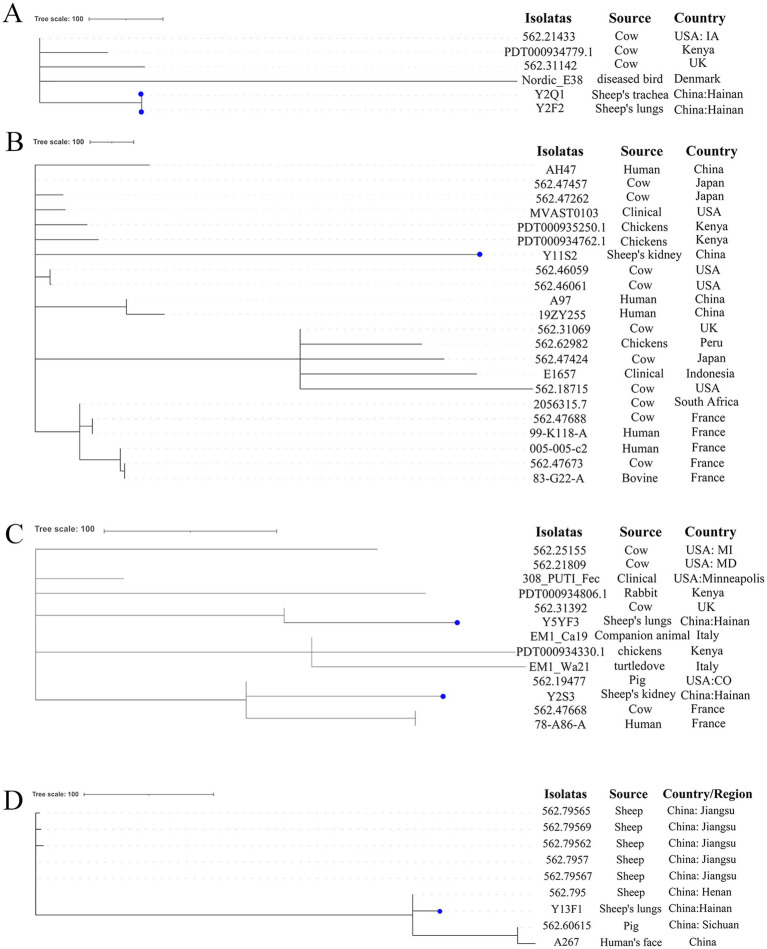
The phylogenetic analysis results of *E. coli* in this study and *E. coli* with the same genotypes in the pubMLST database based on cgMLST. **(A–D)** Separately represent the strains of ST1146, ST1308, ST2521 and ST971 strains in pubMLST database, the dots represent the strains in this study.

Genotypes of *E. coli* isolated in this study were compared to the pubMLST database. We discovered that *E. coli* with the same genotype as Y2F2 has only been reported in cows from the USA, UK, and Kenya, and in birds from Denmark (Figure 4A). However, *E. coli* with the same genotype as Y11S2 in this study exhibited a broader range of sources, including healthy individuals’ feces in China and France; clinical patients in the USA; cows from the USA, UK, Japan, and France, and chickens from Kenya and Peru (Figure 4B). *E. coli* with the same genotypes as Y5YF3 and Y2S3 have been found in clinical patients in the United States; feces from healthy people in France; cows in the USA, UK, and France; and pets, pigs, rabbits, and other animals in various countries (Figure 4C). *E. coli* with the Y2P1 genotype has only been reported in cows from the UK while the Y3X1 genotype was found only in individuals from China and Kenya (Supplementary Table S2). No reports of *E. coli* with the same genotypes as Y2XX1 and Y2P2 were found (Supplementary Table S2). The aforementioned *E. coli* genotypes were initially discovered and reported in sheep. Similarly, *E. coli* with the Y13F1 genotype in this study was primarily found in sheep, with a few individual cases detected in pigs and healthy people in China (Figure 4D).

### Antimicrobial susceptibility assay

**Figure 5:**
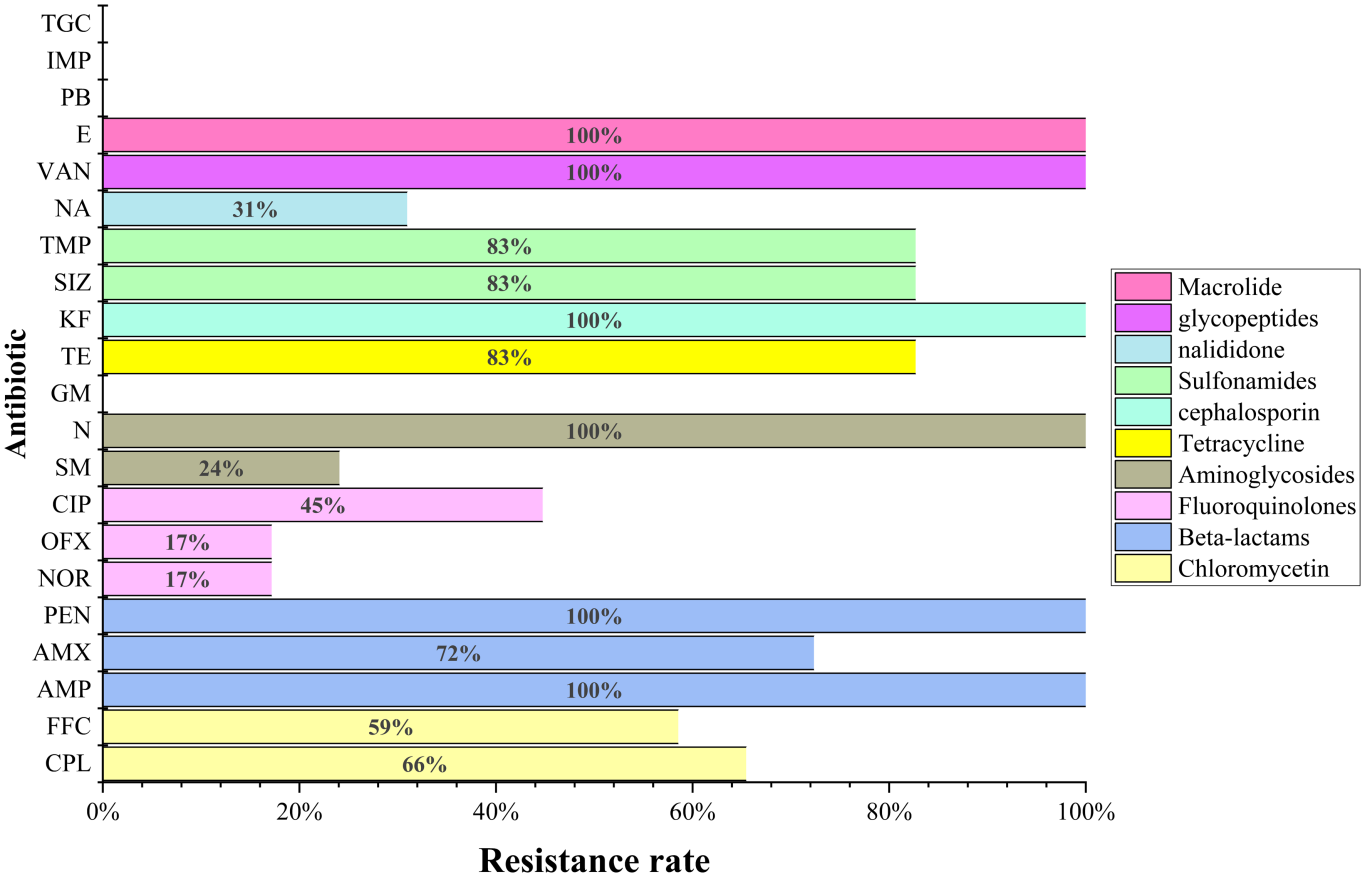
The results of antimicrobial susceptibility tests of the 29 *E. coli* in this study. AMX (amoxicillin), PEN (penicillin), IMP (imipenem), GM (gentamicin), TGC (tigecycline), VAN (vancomycin).

Twenty-one antibiotics were used in susceptibility tests, with isolates showing resistance to at least six and up to sixteen antibiotics (Figure 5 and Table 2). The total resistance rates were 80% to tetracycline and sulfonamides (sulfamethoxazole and trimethoprim), 72% to amoxicillin, 66% to chloramphenicol, 59% to florfenicol, 45% to ciprofloxacin, 31% to nalidixic acid, 24% to streptomycin, and 17% to norfloxacin and ofloxacin. High or intermediate susceptibility was observed for four drugs (polymyxin B, imipenem, tigecycline, and gentamicin) by all isolates. On the other hand, all isolates were resistant to six agents (penicillin, ampicillin, cephalothin, neomycin, erythromycin, and vancomycin) (Figure 5).

**Table 2:** Antimicrobial resistance profile of *E. coli* from dead sheep.

Isolates	Antimicrobial susceptibility pattern		
	Susceptible	Intermediate	Resistance
Y2Q1	PB-IMP-NOR-OFX-GM-NA-TGC	AMX	CPL-FFC-AMP-PEN-CIP-SM-N-TE-KF-SIZ-TMP-VAN-E
Y2XS	PB-FFC-NOR-OFX-SM-GM-NA-TGC	IMP	CPL-AMP-AMX-PEN-CIP-N-TE-KF-SIZ-TMP-VAN-E
Y2F2	PB-FFC-NOR-SM-GM-TGC	IMP-AMX-OFX-NA	CPL-AMP-PEN-CIP-N-TE-KF-SIZ-TMP-VAN-E
Y2X3	PB-IMP-NOR-OFX-GM-TGC	CIP-NA	CPL-FFC-AMP-AMX-PEN-SM-N-TE-KF-SIZ-TMP-VAN-E
Y2S1	PB-IMP-FFC-NOR-OFX-SM-GM-TGC	CIP-N	CPL-AMP-AMX-PEN-TE-KF-SIZ-TMP-NA-VAN-E
Y2XX1	CPL-FFC-NOR-OFX-GM-NA-TGC	PB-IMP-CIP-SM	AMP-AMX-PEN-N-TE-KF-SIZ-TMP-VAN-E
Y2P3	PB-CPL-NOR-OFX-GM-TGC	IMP-SM-NA	FFC-AMP-AMX-PEN-CIP-N-TE-KF-SIZ-TMP-VAN-E
Y2G1	PB-IMP-NOR-OFX-GM-TGC	CIP-SM-NA	CPL-FFC-AMP-AMX-PEN-N-TE-KF-SIZ-TMP-VAN-E
Y2G3	PB-IMP-NOR-OFX-GM-NA-TGC	CPL	FFC-AMP-AMX-PEN-CIP-SM-N-TE-KF-SIZ-TMP-VAN-E
Y2P2	PB-IMP-CPL-FFC-NOR-OFX-GM-TGC	AMX-SM-NA	AMP-PEN-CIP-N-TE-KF-SIZ-TMP-VAN-E
Y2Q3	PB-IMP-CPL-FFC-NOR-OFX-SM-GM-TGC	AMX-CIP-N-NA	AMP-PEN-TE-KF-SIZ-TMP-VAN-E
Y2XX2	PB-IMP-CPL-FFC-NOR-OFX-GM-TGC	AMX-CIP-SM-N-NA	AMP-PEN-TE-KF-SIZ-TMP-VAN-E
Y2P1	PB-IMP-CPL-FFC-NOR-OFX-GM-SIZ-TMP-NA-TGC	AMP-CIP-SM-TE	AMX-PEN-N-KF-VAN-E
Y2S3	PB-IMP-NOR-OFX-GM-NA-TGC	CIP-SM	CPL-FFC-AMP-AMX-PEN-N-TE-KF-SIZ-TMP-VAN-E
Y2X4	IMP-NOR-OFX-SM-GM	PB-CIP-NA	CPL-FFC-AMP-AMX-PEN-N-TE-KF-SIZ-TMP-TGC-VAN-E
Y2X1	PB-IMP-NOR-GM-NA-TGC	OFX-CIP-SM-KF	CPL-FFC-AMP-AMX-PEN-N-TE-SIZ-TMP-VAN-E
Y3G1 Y3Q3 Y3X1 Y3YF1 Y3ZF5	PB-TGC	IMP-SM-GM	CPL-FFC-AMP-AMX-PEN-NOR-OFX-CIP-N-TE-KF-SIZ-TMP-NA-VAN-E
Y5P1 Y5YF3 Y5ZF1	PB-CPL-FFC-NOR-OFX-SM-GM-SIZ-TMP-NA-TGC	IMP-CIP-TE-TE	AMP-AMX-PEN-N-KF-VAN-E
Y11S1 Y11S2	PB-IMP-NOR-TGC	OFX-CIP-GM	CPL-FFC-AMP-AMX-PEN-SM-N-TE-KF-SIZ-TMP-NA-VAN-E
Y12F1 Y12G1	PB-IMP-NOR-OFX-GM-TGC	AMX-NA	CPL-FFC-AMP-PEN-CIP-SM-N-TE-KF-SIZ-TMP-VAN-E
Y13F1	PB-IMP-CPL-FFC-NOR-OFX-GM-TE-SIZ-TMP-TGC	AMX-CIP-SM-N	AMP-PEN-KF-NA-VAN-E

### Detection of antimicrobial resistance genes

A total of ten antimicrobial resistance genes (ARGs) or their variants (such as *aad*A1, *aad*A5, and *aad*A8 of *aad*As) were detected by PCR among the 21 isolates of *E. coli* (Table 3). The number of ARGs harbored by each isolate varied, ranging from 2 (observed in 1 isolate from sheep Y2) to 8 (observed in 6 isolates: 4 from sheep Y2 and 2 from sheep Y11). However, no single ARG was consistently present in all isolates. The frequencies, from high to low, were 89.7% (26/29) for *bla*_TEM_ and *tet*A, 82.8% (24/29) for *bla*_EC_, 72.4% (21/29) for *dfr*A, 65.5% (19/29) for *sul*, 55.2% (16/29) for *qnr*S, 48.3% (14/29) for *aad*A, 41.4% (12/29) for *flo*R, 34.5% (10/29) for *aph*, and 17.2% (5/29) for *amp*C (*bla*_CMY_). Furthermore, at least one of the three ARGs targeting β-lactamases was found in all 29 isolates.

**Table 3:** Detection of antimicrobial resistance genes in *E. coli.*

Class	Aminoglycosides		β-lactamases			Diaminopyrimidines	Fluoroquinolones	Phenicols	Sulfonamide	Tetracycline	Total number of ARGs
Resistance genes	*aad*A	*aph*	*amp*C	*bla*_EC_	bla_TEM_	*dfr*A	QnrS	*flo*R	*sul*	*tet*A	
Antibiotics	SM	N etc.	KF etc.		AMP	TMP	CIP, NOR etc.	FFC, CPL etc.	SIZ etc.	TE	
Mechanism	APH catalyzes the transfer of the gamma-phosphoryl group from ATP to aminoglycoside antibiotics such as kanamycin, streptomycin, neomycin, and gentamicin, among others					Antimicrobial target alteration	Antimicrobial target protection	Antimicrobial efflux	Antimicrobial target alteration	Antimicrobial efflux	
Y2Q1	+			+	+		+	+	+	+	7
Y2XS	+			+	+	+	+		+	+	7
Y2F2	+			+	+	+	+		+	+	7
Y2X3	+	+		+	+	+	+	+		+	8
Y2S1				+	+						2
Y2XX1		+		+	+				+	+	5
Y2P3		+		+	+			+	+	+	6
Y2G1				+	+		+				3
Y2G3		+		+	+		+	+	+	+	7
Y2P2	+			+	+	+	+				5
Y2Q3	+			+	+	+	+	+		+	7
Y2XX2	+			+	+	+	+	+	+	+	8
Y2P1				+	+				+	+	4
Y2S3	+	+		+	+	+	+		+	+	8
Y2X4				+	+		+	+	+	+	6
Y2X1	+	+		+	+	+	+		+	+	8
Y3G1	+		+		+	+				+	5
Y3Q3	+		+		+	+				+	5
Y3X1	+		+		+	+				+	5
Y3YF1	+		+		+	+				+	5
Y3ZF5	+		+		+	+				+	5
Y5P1				+	+	+			+	+	5
Y5YF3				+	+	+			+	+	5
Y5ZF1				+	+	+			+	+	5
Y11S1		+		+	+	+	+	+	+	+	8
Y11S2		+		+	+	+	+	+	+	+	8
Y12F1		+		+		+	+	+	+	+	7
Y12G1		+		+		+	+	+	+	+	7
Y13F1				+		+		+	+	+	5
Detection rate	48.28%(14/29)	34.48%(10/29)	17.24% (5/29)	82.76% (24/29)	89.66% (26/29)	72.41% (21/29)	55.17% (16/29)	41.38% (12/29)	65.52% (19/29)	89.66% (26/29)	

Crosses indicate the presence of a gene. SM (streptomycin), N (neomycin), KF (cephalothin), AMP (ampicillin), TMP (trimethoprim), CIP (ciprofloxacin), NOR (norfloxacin), FFC (florfenicol), CPL (chloramphenicol), OFX (ofloxacin), NA (nalidixic acid), E (erythromycin), PB (polymyxin B), SIZ (sulfamethoxazole), TE (tetracycline).

## Discussion

In recent years, an increasing number of studies have reported *E. coli* infections from non-intestinal organs of sick and dead sheep, raising concerns about animal health risks. Supporting these concerns, Kjelstrup et al. (2013) isolated *E. coli* from vital organs of lambs that died from septicemia and Singh et al. (2019) found it in the hearts and lungs of sheep and goats suffering from pneumonia or septicemia. Our study, similar to previous findings, isolated *E. coli* from various organs (liver, heart, kidneys, and lungs) of sheep with pneumonia. Strikingly, we found a rare case where *E. coli* was identified in multiple vital organs of a single sheep (sheep 1). We postulate that the systemic infection is likely to be related to the high virulency of the pathogens and low immunity of the sheep 1. Furthermore, not only sheep 1 but also other sheep in this study exhibit the phenomenon of mixed infection with multiple strains. This could be related to the fact that the sheep on this farm are introduced from inland China to Hainan Province. As they are not acclimatized to the tropical climate of Hainan Province and are highly vulnerable to attacks by local pathogens. As described in previous studies, pathogens have a very strong ability to spread across regions (Cheng et al., 2015). Coupled with the inherent pathogens carried by the sheep themselves upon introduction, it is very likely to lead to the risk of mixed infection with multiple strains for these sheep. In the field of veterinary science, understanding such factors is crucial for formulating effective prevention and control strategies.

It is noteworthy that as time goes on, the number of *E. coli* isolated at different times decreases in turn (Table 1). This is hypothesized to be related to two factors. On one hand, the sheep introduced from mainland China to Hainan Province become more adapted to the local climate and show increased resistance to pathogens over time. On the other hand, at the initial stage of the epidemic outbreak, the farm employed antibiotic treatment, which eliminated most of the pathogens sensitive to antibiotics.

Subsequent identification shows that three isolates belonged to ExPEC, which were isolated from the spleen (one strain) and lungs (two strains) of sheep 1 (Table 1). Genotyping and serotyping are both essential methods within current bacterial typing systems. However, the correlation between the two for bacterial classification is infrequently reported. In our study, we employed the two prevailing *E. coli* MLST typing methods (the Achtman scheme and Pasteur scheme) to investigate the STs. We discovered a certain correlation between STs and serotypes. However, the STs and serotypes could not be matched one by one using a single MLST scheme. Strains belonging to the same serotype could exhibit different STs. This is because strains within the same serotype group might have variations in the specific genes analyzed by the chosen MLST scheme. Since they target different conserved genes, combining both schemes can circumvent this issue (Figure 2). Further optimization of this combined MLST scheme holds promise a more suitable typing method for *E. coli* that would be advantageous for the prevention and management of pathogenic *E. coli*.

This study identified a concerning number of virulence factors (25) and various combinations (8) within the isolates (Figure 2). A number of previous studies have suggested that the diversity and combination of virulence factors may intensify the challenges of infection control (Liu et al., 2015). In this study, strains with varying combinations of virulence factors demonstrated distinct lethality and pneumonia (Figure 3), with Y13F1 the most lethal strain (Figure 3A). This may be attributed to its possession of the highest number of virulence factors, including two related to iron uptake. Studies have shown that strains carrying such virulence factors possess greater virulence and environmental adaptability (Lemos and Balado, 2020), as well as a higher propensity to cause urinary tract infections and even septicemia (Frick-Cheng et al., 2022). Interestingly, isolate Y3X1, despite carrying the lowest number of virulence factors, caused the most severe lung inflammatory response in the mouse infection model (Figure 3D). This phenomenon might be explained by its possession of specific virulence factors, Tia and Hek, which are related to invasins activity. Maciel et al. (2017) demonstrated that this virulence factor plays a crucial role in avian septicemia caused by *E. coli*. ExPEC is a primary pathogen of hospital-acquired infections, frequently causing septicemia and urinary tract infections and exhibiting high pathogenicity (Shulman et al., 2018). Consistent with previous findings, the ExPEC (Y5YF3) isolate also exhibited strong pathogenicity, and causing weight loss, lung inflammation, and severe melanization in the spleen (Figures 3B,C).

While the presence of drug-resistance genes does not guarantee the development of antimicrobial resistance, it can slightly increase the chances of bacteria becoming resistant. This phenomenon adds to the complexity of treatment and prevention. All isolates, with the exception of “Y2S1” and “Y2G1,” carried at least four types of ARGs (Table 3). This number is significantly higher than previous studies. Furlan et al. (2019) isolated *E. coli* from sheep feces primarily carried one or two types of ARGs. Moreover, Gencay (2014) isolated 24 strains of *E. coli* O157 from sheep having only 3 strains with 3 types of ARGs. In addition, the prevalence of certain ARGs found in our study was much higher than previously reported in sheep. For instance, the *bla*TEM gene, one of the most prevalent ESBLs genes in *E. coli*, can lead to bacterial resistance to third-generation cephalosporin antibiotics (Gundran et al., 2019). Among the 17 *E. coli* strains isolated from sheep by Gupta et al. (2022), the prevalence of *bla*TEM was 35%, which was significantly lower than our study (89.66%). *TetA* and *tetB* are the most important ARGs for tetracycline resistance in *E. coli* isolated from different animals (Bryan et al., 2004). In the 19 strains of *E. coli* isolated from sheep reported by Furlan et al. (2019), the detection rate of *tetA* or *tetB* was 36.84%, which is also much lower than our study (89.66%).

All isolates were categorized as MDR bacteria showing resistance to at least 6 out of 21 antibiotics tested, and while 75.86% (22 isolates) were resistant to over 10 antibiotics. Of note, 5 *E. coli* strains isolated from various organs of “sheep 2” were entirely resistant to 15 of the 21 antibiotics, intermediately resistant to 4, and only fully susceptible to polymyxin B and tetracycline (Table 2). Furthermore, all isolates were completely resistant to 6 of the 21 antibiotics, and over half of the isolates were completely resistant to 6 of the remaining 15 antibiotics (Figure 5). This finding highlights a significant increase in antimicrobial resistance compared to previous studies. In a study by Gencay (2014), only four out of 24 *E. coli* isolates from sheep showed intermediate resistance to cephalosporin antibiotics, with none exhibiting complete resistance to the remaining. The rate of complete resistance to antibiotics in *E. coli* isolated from sheep in Bangladesh by Gupta et al. (2022) did not exceed 50% for 10 tested drugs. Antimicrobial resistance occurs primarily due to misuse and overuse. Consequently, farms should be counseled to employ rational antibiotic usage to preclude abuse and misuse. Concurrently, relevant departments can provide training on “rational antibiotic use.”

Multiple previous studies have demonstrated that *E. coli* isolated from poultry and birds (particularly ExPEC) possess zoonotic potential (Hu et al., 2022; Xia et al., 2022; Skarżyńska et al., 2021). Based on the virulence overlap between *E. coli* isolated from sheep and enteropathogenic *E. coli*, Zhao J. Y. et al. (2021) and Zhao X. et al. (2021) suggested that *E. coli* derived from sheep may also have zoonotic potential. According to the Achtman scheme, all isolates were categorized into eight sequence types. Further analysis of strain information from pubMLST databases and cgMLST, excluding Y13F1, which is a common genotype in sheep, showed the strains were first reported in sheep. In previous studies, these strains were mainly found in human feces, patients in hospitals, cows (Figure 4 and Supplementary Table S2), and other animals such as (Zhang et al., 2021), pigs (Hsueh et al., 2020), and horses (Elias et al., 2019). A multi-host source of strains of the same genotype indicating interspecies transmission of virulent and multidrug-resistant isolates from sheep to humans and other farmed animals or from humans and other farmed animals to sheep is highly possible. Most importantly, sheep are a vital source of meat globally, including China. Isolating such highly virulent *E. coli* strains from sheep poses a serious risk to human health.

Mouse infection models have a crucial role in assessing the public health safety risks posed by highly virulent pathogens to mammals (Sarkar and Heise, 2019). Mouse infection model results reveal that strains Y13F1 and Y3YF3 cause the most severe pneumonia and highest mortality in mice. The relationship between the ability to cause pneumonia or death and the phenotypes/genotypes of these strains merits further study.

As an international free-trade port, Hainan Island’s frequent trade exchanges make it highly vulnerable to biosecurity threats from foreign pathogens. The multidrug-resistant and potentially zoonotic *E. coli* strains identified in this study underscore the importance of the One Health concept (Atlas, 2013). Collaborative efforts among different departments are essential to strengthen quarantine measures. This will prevent the introduction of highly virulent, multidrug-resistant, and zoonotic pathogens to Hainan Island, along with the spread of invasive species.

## Conclusion

Sheep are a vital source of meat for human consumption. The occurrence of pneumonia not only jeopardizes the health of sheep flocks but also threatens the economic stability of farms. Our findings confirmed the significant role of *E. coli* in causing suppurative pneumonia in sheep. Diseased sheep have become a novel reservoir of highly virulent and multidrug-resistant *E. coli* strains with the potential to transmit across species and pose a zoonotic threat. We also characterized the diversity of sequence types, virulence factor combinations, and antimicrobial resistance profiles of *E. coli* within sheep breeding populations affected by pneumonia in Hainan Province. These results have significant implications for developing effective control measures to prevent the spread of this pathogen within sheep farms and potentially to humans.

## Data Availability

The datasets presented in this study can be found in online repositories. The names of the repository/repositories and accession number(s) can be found at: https://www.ncbi.nlm.nih.gov/, PRJNA1142750.

## Footnotes

^1^https://www.servicebio.cn/

^2^http://www.eucast.org/clinical_breakpoints/

^3^http://www.mgc.ac.cn/cgi-bin/VFs/v5/main.cgi

^4^https://pubmlst.org/

^5^https://itol.embl.de/

^6^http://genomicepidemiology.org/

## References

[ref1] Aiello S. E. Moses M. A. (2016). The Merck veterinary manual. 11th Edn. New Jersey, USA: Wiley.

[ref2] Alley M. R. Ionas G. Clarke J. K. (1999). Chronic non-progressive pneumonia of sheep in New Zealand—a review of the role of *Mycoplasma ovipneumoniae*. N. Z. Vet. J. 47, 155–160. doi: 10.1080/00480169.1999.36135, PMID: 16032095

[ref3] Atlas R. M. (2013). One Health: its origins and future. Curr. Top. Microbiol. Immunol. 365, 1–13. doi: 10.1007/82_2012_223 22527177

[ref4] Besser T. E. Frances Cassirer E. Highland M. A. Wolff P. Justice-Allen A. Mansfield K.. (2013). Bighorn sheep pneumonia: sorting out the cause of a polymicrobial disease. Prev. Vet. Med. 108, 85–93. doi: 10.1016/j.prevetmed.2012.11.018, PMID: 23253148

[ref5] Bryan A. Shapir N. Sadowsky M. J. (2004). Frequency and distribution of tetracycline resistance genes in genetically diverse, nonselected, and nonclinical *Escherichia coli* strains isolated from diverse human and animal sources. Appl. Environ. Microbiol. 70, 2503–2507. doi: 10.1128/AEM.70.4.2503-2507.2004, PMID: 15066850 PMC383146

[ref6] Cheng C. Jun Q. Qingling M. Zhengxiang H. Yu M. Xuepeng C.. (2015). Serological and molecular survey of sheep infected with *Mycoplasma ovipneumoniae* in Xinjiang, China. Trop. Anim. Health Prod. 47, 1641–1647. doi: 10.1007/s11250-015-0908-2, PMID: 26315151

[ref7] Clermont O. Christenson J. K. Denamur E. Gordon D. M. (2013). The Clermont *Escherichia coli* phylo-typing method revisited: improvement of specificity and detection of new phylo-groups. Environ. Microbiol. Rep. 5, 58–65. doi: 10.1111/1758-2229.12019, PMID: 23757131

[ref8] Clermont O. Gordon D. Denamur E. (2015). Guide to the various phylogenetic classification schemes for *Escherichia coli* and the correspondence among schemes. Microbiology 161, 980–988. doi: 10.1099/mic.0.000063 25714816

[ref9] Desvars-Larrive A. Vogl A. E. Puspitarani G. A. Yang L. Joachim A. Käsbohrer A. (2024). A One Health framework for exploring zoonotic interactions demonstrated through a case study. Nat. Commun. 15:5650. doi: 10.1038/s41467-024-49967-7, PMID: 39009576 PMC11250852

[ref11] Edgar R. C. (2018). Updating the 97% identity threshold for 16S ribosomal RNA OTUs. Bioinformatics 34, 2371–2375. doi: 10.1093/bioinformatics/bty113, PMID: 29506021

[ref12] Elias L. Gillis D. C. Gurrola-Rodriguez T. Jeon J. H. Lee J. H. Kim T. Y.. (2019). The occurrence and characterization of extended-spectrum-beta-lactamase-producing *Escherichia coli* isolated from clinical diagnostic specimens of equine origin. Animals 10:28. doi: 10.3390/ani10010028, PMID: 31877788 PMC7022413

[ref13] Frick-Cheng A. E. Sintsova A. Smith S. N. Pirani A. Snitkin E. S. Mobley H. L. T. (2022). Ferric citrate uptake is a virulence factor in uropathogenic *Escherichia coli*. mBio 13:e0103522. doi: 10.1128/mbio.01035-22, PMID: 35546538 PMC9239202

[ref14] Furlan J. P. R. Gallo I. F. L. de Campos A. C. L. P. Navarro A. Kobayashi R. K. T. Nakazato G.. (2019). Characterization of non-O157 Shiga toxin-producing *Escherichia coli* (STEC) obtained from feces of sheep in Brazil. World J. Microbiol. Biotechnol. 35:134. doi: 10.1007/s11274-019-2712-z, PMID: 31432266

[ref15] Gencay Y. E. (2014). Sheep as an important source of *E. coli* O157/O157:H7 in Turkey. Vet. Microbiol. 172, 590–595. doi: 10.1016/j.vetmic.2014.06.014 25042529

[ref16] Gundran R. S. Cardenio P. A. Villanueva M. A. Sison F. B. Benigno C. C. Kreausukon K.. (2019). Prevalence and distribution of Bla CTX-M, Bla SHV, bla TEM genes in extended-spectrum β-lactamase-producing *E. coli* isolates from broiler farms in the Philippines. BMC Vet. Res. 15:227. doi: 10.1186/s12917-019-1975-9, PMID: 31277658 PMC6612079

[ref17] Gupta M. D. Sen A. Shaha M. Dutta A. Das A. (2022). Occurrence of Shiga toxin-producing *Escherichia coli* carrying antimicrobial resistance genes in sheep on smallholdings in Bangladesh. Vet. Med. Sci. 8, 2616–2622. doi: 10.1002/vms3.935, PMID: 36095131 PMC9677359

[ref18] Hsueh S. C. Lai C. C. Huang Y. T. Liao C. H. Chiou M. T. Lin C. N.. (2020). Molecular evidence for intra-and inter-farm spread of porcine mcr-1-carrying *Escherichia coli* in Taiwan. Front. Microbiol. 11:1967. doi: 10.3389/fmicb.2020.01967, PMID: 32973713 PMC7468466

[ref19] Hu J. Afayibo D. J. A. Zhang B. Zhu H. Yao L. Guo W.. (2022). Characteristics, pathogenic mechanism, zoonotic potential, drug resistance, and prevention of avian pathogenic *Escherichia coli* (APEC). Front. Microbiol. 13:1049391. doi: 10.3389/fmicb.2022.1049391, PMID: 36583051 PMC9793750

[ref20] Humphries R. Bobenchik A. M. Hindler J. A. Schuetz A. N. (2021). Overview of changes to the Clinical and Laboratory Standards Institute *Performance Standards for Antimicrobial Susceptibility Testing*, M100, 31st edition. J. Clin. Microbiol. 59:e0021321. doi: 10.1128/JCM.00213-21, PMID: 34550809 PMC8601225

[ref21] Jia Y. Mao W. Liu B. Zhang S. Cao J. Xu X. (2022). Study on the drug resistance and pathogenicity of *Escherichia coli* isolated from calf diarrhea and the distribution of virulence genes and antimicrobial resistance genes. Front. Microbiol. 13:992111. doi: 10.3389/fmicb.2022.992111, PMID: 36620061 PMC9815963

[ref22] Joensen K. G. Tetzschner A. M. Iguchi A. Aarestrup F. M. Scheutz F. (2015). Rapid and easy *in silico* serotyping of *Escherichia coli* isolates by use of whole-genome sequencing data. J. Clin. Microbiol. 53, 2410–2426. doi: 10.1128/JCM.00008-15, PMID: 25972421 PMC4508402

[ref23] Johnson J. R. Murray A. C. Gajewski A. Sullivan M. Snippes P. Kuskowski M. A.. (2003). Isolation and molecular characterization of nalidixic acid-resistant extraintestinal pathogenic *Escherichia coli* from retail chicken products. Antimicrob. Agents Chemother. 47, 2161–2168. doi: 10.1128/AAC.47.7.2161-2168.2003, PMID: 12821463 PMC161843

[ref24] Kjelstrup C. K. Arnesen L. P. Granquist E. G. L'Abée-Lund T. M. (2013). Characterization of *Escherichia coli* O78 from an outbreak of septicemia in lambs in Norway. Vet. Microbiol. 166, 276–280. doi: 10.1016/j.vetmic.2013.05.004, PMID: 23768929

[ref25] Kuraz Abebe B. (2022). A review of the potential and constraints for crossbreeding as a basis for goat production by smallholder farmers in Ethiopia. Bull. Natl. Res. Cent. 46:80. doi: 10.1186/s42269-022-00763-7

[ref26] Lemos M. L. Balado M. (2020). Iron uptake mechanisms as key virulence factors in bacterial fish pathogens. J. Appl. Microbiol. 129, 104–115. doi: 10.1111/jam.14595, PMID: 31994331

[ref27] Liu X. Thungrat K. Boothe D. M. (2015). Multilocus sequence typing and virulence profiles in uropathogenic *Escherichia coli* isolated from cats in the United States. PLoS One 10:e0143335. doi: 10.1371/journal.pone.0143335, PMID: 26587840 PMC4654559

[ref28] Long N. Deng J. Qiu M. Zhang Y. Wang Y. Guo W.. (2022). Inflammatory and pathological changes in *Escherichia coli* infected mice. Heliyon 8:e12533. doi: 10.1016/j.heliyon.2022.e12533, PMID: 36643320 PMC9834738

[ref29] Maciel J. F. Matter L. B. Trindade M. M. Camillo G. Lovato M. de Ávila B. S.. (2017). Virulence factors and antimicrobial susceptibility profile of extraintestinal *Escherichia coli* isolated from an avian colisepticemia outbreak. Microb. Pathog. 103, 119–122. doi: 10.1016/j.micpath.2016.12.020, PMID: 28012984

[ref31] Maluta R. P. Logue C. M. Casas M. R. Meng T. Guastalli E. A. Rojas T. C.. (2014). Overlapped sequence types (STs) and serogroups of avian pathogenic (APEC) and human extra-intestinal pathogenic (ExPEC) *Escherichia coli* isolated in Brazil. PLoS One 9:e105016. doi: 10.1371/journal.pone.0105016, PMID: 25115913 PMC4130637

[ref32] Manges A. R. Harel J. Masson L. Edens T. J. Portt A. Reid-Smith R. J.. (2015). Multilocus sequence typing and virulence gene profiles associated with *Escherichia coli* from human and animal sources. Foodborne Pathog. Dis. 12, 302–310. doi: 10.1089/fpd.2014.1860, PMID: 25774654

[ref34] Mekibib B. Mikir T. Fekadu A. Abebe R. (2019). Prevalence of pneumonia in sheep and goats slaughtered at Elfora Bishoftu export abattoir, Ethiopia: a pathological investigation. J. Vet. Med. 2019, 5169040–5169010. doi: 10.1155/2019/5169040, PMID: 31396540 PMC6668555

[ref35] Mellata M. (2013). Human and avian extraintestinal pathogenic *Escherichia coli*: infections, zoonotic risks, and antibiotic resistance trends. Foodborne Pathog. Dis. 10, 916–932. doi: 10.1089/fpd.2013.1533, PMID: 23962019 PMC3865812

[ref36] Poolman J. T. Wacker M. (2016). Extraintestinal pathogenic *Escherichia coli*, a common human pathogen: challenges for vaccine development and Progress in the field. J. Infect. Dis. 213, 6–13. doi: 10.1093/infdis/jiv429 26333944 PMC4676548

[ref37] Rahsan Y. Nihat Y. Bestami Y. Adnan A. Nuran A. (2018). Histopathological, immunohistochemical, and parasitological studies on pathogenesis of *Coenurus cerebralis* in sheep. J. Vet. Res. 62, 35–41. doi: 10.2478/jvetres-2018-0005, PMID: 29978125 PMC5957459

[ref38] Rehman M. N. U. Dawar F. U. Zeng J. Fan L. Feng W. Wang M.. (2023). Complete genome sequence analysis of *Edwardsiella tarda* SC002 from hatchlings of Siamese crocodile. Front. Vet. Sci. 10:1140655. doi: 10.3389/fvets.2023.1140655, PMID: 36968469 PMC10034365

[ref39] Rehman M. N. U. Yu W. Pan J. Han Y. Yang N. Wang X.. (2021). Histological and molecular characterization of *Edwardsiella tarda* infection in Siamese crocodile (*Crocodylus siamensis*) hatchlings. Aquaculture 535:736367. doi: 10.1016/j.aquaculture.2021.736367

[ref41] Rodriguez-Siek K. E. Giddings C. W. Doetkott C. Johnson T. J. Fakhr M. K. Nolan L. K. (2005). Comparison of *Escherichia coli* isolates implicated in human urinary tract infection and avian colibacillosis. Microbiology 151, 2097–2110. doi: 10.1099/mic.0.27499-0, PMID: 15942016

[ref42] Russo T. A. Johnson J. R. (2003). Medical and economic impact of extraintestinal infections due to *Escherichia coli*: focus on an increasingly important endemic problem. Microbes Infect. 5, 449–456. doi: 10.1016/s1286-4579(03)00049-2, PMID: 12738001

[ref43] Sarkar S. Heise M. T. (2019). Mouse models as resources for studying infectious diseases. Clin. Ther. 41, 1912–1922. doi: 10.1016/j.clinthera.2019.08.010, PMID: 31540729 PMC7112552

[ref44] Scott P. R. (2011). Treatment and control of respiratory disease in sheep. Vet. Clin. North Am. Food Anim. Pract. 27, 175–186. doi: 10.1016/j.cvfa.2010.10.016 21215901

[ref45] Shulman A. Yair Y. Biran D. Sura T. Otto A. Gophna U.. (2018). The *Escherichia coli* type III secretion system 2 has a global effect on cell surface. mBio 9, e01070–e01018. doi: 10.1128/mBio.01070-18, PMID: 29970469 PMC6030553

[ref46] Singh F. Sonawane G. G. Kumar J. Dixit S. K. Meena R. K. Tripathi B. N. (2019). Antimicrobial resistance and phenotypic and molecular detection of extended-spectrum ß-lactamases among extraintestinal *Escherichia coli* isolated from pneumonic and septicemic sheep and goats in Rajasthan, India. Turk. J. Vet. Anim. Sci. 43, 754–760. doi: 10.3906/vet-1905-1

[ref47] Skarżyńska M. Zaja C. M. Bomba A. Bocian Ł. Kozdruń W. Polak M.. (2021). Antimicrobial resistance glides in the sky-free-living birds as a reservoir of resistant *Escherichia coli* with zoonotic potential. Front. Microbiol. 12:656223. doi: 10.3389/fmicb.2021.656223, PMID: 33897669 PMC8062882

[ref48] Tivendale K. A. Logue C. M. Kariyawasam S. Jordan D. Hussein A. Li G.. (2010). Avian-pathogenic *Escherichia coli* strains are similar to neonatal meningitis *E. coli* strains and are able to cause meningitis in the rat model of human disease. Infect. Immun. 78, 3412–3419. doi: 10.1128/IAI.00347-10, PMID: 20515929 PMC2916289

[ref49] Xia F. Jiang M. Wen Z. Wang Z. Wang M. Xu Y.. (2022). Complete genomic analysis of ST117 lineage extraintestinal pathogenic *Escherichia coli* (ExPEC) to reveal multiple genetic determinants to drive its global transmission: ST117 *E. coli* as an emerging multidrug-resistant foodborne ExPEC with zoonotic potential. Transbound. Emerg. Dis. 69, 3256–3273. doi: 10.1111/tbed.14678, PMID: 35945191

[ref50] Young L. Sung J. Stacey G. Masters J. R. (2010). Detection of mycoplasma in cell cultures. Nat. Protoc. 5, 929–934. doi: 10.1038/nprot.2010.43 20431538

[ref51] Zhang Y. Kuang X. Liu J. Sun R. Y. Li X. P. Sun J.. (2021). Identification of the plasmid-mediated colistin resistance gene mcr-1 in *Escherichia coli* isolates from migratory birds in Guangdong, China. Front Microbiol. 12:755233. doi: 10.3389/fmicb.2021.755233, PMID: 34745062 PMC8567052

[ref52] Zhao J. Y. Du Y. Z. Song Y. P. Zhou P. Chu Y. F. Wu J. Y. (2021). Investigation of the prevalence of *Mycoplasma Ovipneumoniae* in Southern Xinjiang, China. J. Vet. Res. 65, 155–160. doi: 10.2478/jvetres-2021-0021, PMID: 34250299 PMC8256467

[ref53] Zhao L. Gao S. Huan H. Xu X. Zhu X. Yang W.. (2009). Comparison of virulence factors and expression of specific genes between uropathogenic *Escherichia coli* and avian pathogenic *E. coli* in a murine urinary tract infection model and a chicken challenge model. Microbiology 155, 1634–1644. doi: 10.1099/mic.0.024869-0 19372154

[ref54] Zhao X. Lv Y. Adam F. E. A. Xie Q. Wang B. Bai X.. (2021). Comparison of antimicrobial resistance, virulence genes, phylogroups, and biofilm formation of *Escherichia coli* isolated from intensive farming and free-range sheep. Front. Microbiol. 12:699927. doi: 10.3389/fmicb.2021.699927, PMID: 34394043 PMC8362090

