# Supplementary material for: Emergence of highly virulent and multidrug-resistant Escherichia coli in breeding sheep with pneumonia, Hainan Province, China
Source: Front Microbiol. 2024 Oct 23;15:1479759. doi: 10.3389/fmicb.2024.1479759 (PMC11539166; doi:10.3389/fmicb.2024.1479759)
Supplement: Supplementary file 1 [file Table_1.docx]

**Table S1: PCR prime and conditions used in the study**

1 Primers for 16S rRNA amplification

| Gene | Prime name | Primer sequence （5’ – 3’） | Temperature of annealing（℃） | Length of PCR products (bp) |
| --- | --- | --- | --- | --- |
| 16S rDNA | 27F | AGAGTTTGATCAGGCTCAG | 55 | 1465 |
|  | 1492R | CGGTTACCTTGTTACGACTT | 55 |  |

2 Primers for identification of ExPEC and non-ExPEC

| Gene | Prime name | Primer sequence （5’ – 3’） | Temperature of annealing（℃） | Length of PCR products (bp) |
| --- | --- | --- | --- | --- |
| *iut*A | *iut*A-F | CAGTATCAGGAATCAGGTAGTCCA | 59 | 674 |
|  | *iut*A-R | CGGTTACCTTGTTACGACTT | 58 |  |
| *pap*A | *pap*A-F | TGGGCATTATTGGCTGACGA | 59 | 470 |
|  | *pap*A-R | TGGCAGTGGTGTCTTTTGGT | 60 |  |
| *kps*MⅡ | *kps*MⅡ-F | GCGCATTTGCTGATACTGTTG | 58 | 270 |
|  | *kps*MⅡ-R | CATCCAGACGATAAGCATGAGCA | 60 |  |
| *pap*C | papC-F | GTGGCAGTATGAGTAATGACCGTTA | 60 | 203 |
|  | *pap*C-R | ATATCCTTTCTGCAGGGATGCAATA | 60 |  |
| *sfa*S | *sfa*S-F | GTCTCTCACCGGATGCCAGAATAT | 62 | 138 |
|  | *sfa*S-R | GCATTACTTCCATCCCTGTCCTG | 61 |  |
| *foc*G | *foc*G-F | AGAGTTTGATCAGGCTCAG | 62 | 369 |
|  | *foc*G-R | CGGTTACCTTGTTACGACTT | 61 |  |
| *dra* | *dra*-F | CCGTCTTGATGGTCAGCAGT | 60 | 457 |
|  | *dra*-R | CCCGCCGTAATCCGTTATCA | 60 |  |
| *afa* | *afa*-F | TCTGGGTATCCCTCAGGTGG | 60 | 576 |
|  | *afa*-R | TCTGATTCCAGCACCGTCAC | 60 |  |

3 Primers for detection of Mycoplasma

3.1 Detection of *Mycoplasma ovipneumoniae*

| Gene | Prime name | Primer sequence （5’ – 3’） | Temperature of annealing（℃） | Length of PCR products (bp) |
| --- | --- | --- | --- | --- |
| 16S rDNA | LMF-F | TGAACGGAATATGTTAGCTT | 52 | 361 |
|  | LMR-R | GACTTCATCCTGCACTCTGT | 57 |  |

3.2 Detection of *Mycoplasma mycoides*

| Gene | Prime name | Primer sequence （5’ – 3’） | Temperature of annealing（℃） | Length of PCR products (bp) |
| --- | --- | --- | --- | --- |
| *lpp*A | *lpp*A-F | ACAAAAAGAAGATATGGTGTTGG | 55 | 717 |
|  | *lpp*A-R | ATCAGGTTTATCCATTGGTTGG | 56 |  |

4 Primers for detection of MLST

4.1 Achtman scheme

| Gene | Prime name | Primer sequence （5’ – 3’） | Temperature of annealing（℃） | Length of PCR products (bp) |
| --- | --- | --- | --- | --- |
| *adk* | *adk*-F | GCGTATCATTCTGCTTGGCG | 60 | 598 |
|  | *adk*-R | CTTGGTGCCGTCAACTTTCG | 60 |  |
| *fum*C | *fum*C-F | CCAGTTCATCTGCTACGCGA | 60 | 656 |
|  | *fumC*-R | TTTCCGCATTTCGACGGAGA | 60 |  |
| *gyr*B | *gyrB* -F | GGCACTTTCACGGAAACGAC | 60 | 658 |
|  | *gyrB* -R | ATAAAGTGTCCGGCGGTCTG | 60 |  |
| *icd* | *icd* -F | AGATGTAACCCCAGCCATGC | 60 | 660 |
|  | *icd* -R | TCGATCAGTTCACCGCCAAA | 60 |  |
| *mdh* | *mdh* -F | ACCCAACTGCCTTCAGGTTC | 60 | 696 |
|  | *mdh* -R | GTAGGCACATTCGACAACGC | 60 |  |
| *pur*A | *pur*A-F | CGCGCTGATGAAAGAGATGA | 58 | 817 |
|  | *pur*A-R | CGCGCTGATGAAAGAGATGA | 60 |  |
| *rec*A | *rec*A-F | CGCATTCGCTTTACCCTGACC | 61 | 734 |
|  | *rec*A-R | TCGTCGAAATCTACGGACCGGA | 63 |  |

4.2 Pasteur scheme

| Gene | Prime name | Primer sequence （5’ – 3’） | Temperature of annealing（℃） | Length of PCR products (bp) |
| --- | --- | --- | --- | --- |
| *trp*A | *trp*A -F | GCTTTCATCGGTTGTACAAA | 57 | 784 |
|  | *trp*A -R | GCTACGAATCTCTGTTTGCC | 54 |  |
| *din*B | *din*B -F | TGAGAGGTGAGCAATGCGTA | 59 | 606 |
|  | *dinB* -R | CGTAGCCCCATCGCTTCCAG | 63 |  |
| *icd*A | *icdA* -F | ATTCGCTTCCCGGAACATTG | 58 | 677 |
|  | *icdA* -R | ATGATCGCGTCACCAAATTC | 56 |  |
| *pab*B | *pab*B -F | AATCCAATATGACCCGCGAG | 57 | 598 |
|  | *pab*B -R | GGTTCCAGTTCGTCGATAAT | 55 |  |
| *pol*B | *pol*B -F | GGCGGCTATGTGATGGATTC | 58 | 616 |
|  | *pol*B -R | GGTTGGCATCAGAAAACGGC | 60 |  |
| *put*P | *put*P -F | CTGTTTAACCCGTGGATTGC | 57 | 506 |
|  | *put*P -R | GCATCGGCCTCGGCAAAGCG | 67 |  |
| *trpB* | *trp*B-F | CACTATATGCTGGGCACCGC | 61 | 632 |
|  | *trp*B-R | CCTCGTGCTTTCAAAATATC | 52 |  |
| *uid*A | *uid*A-F | CATTACGGCAAAGTGTGGGTCAAT | 62 | 658 |
|  | *uid*A-R | CCATCAGCACGTTATCGAATCCTT | 61 |  |

5 Primers for detection of phylo-group

| Gene | Prime name | Primer sequence （5’ – 3’） | Temperature of annealing（℃） | Length of PCR products (bp) |
| --- | --- | --- | --- | --- |
| *chu*A | *chu*A-F | ATGGTACCGGACGAACCAAC | 60 | 288 |
|  | *chu*A-R | TGCCGCCAGTACCAAAGACA | 62 |  |
| *yja*A | *yja*A -F | CAAACGTGAAGTGTCAGGAG | 56 | 211 |
|  | *yja*A -R | AATGCGTTCCTCAACCTGTG | 58 |  |
| *tsp*E4.C2 | *tsp*E4*.*C2 -F | CACTATTCGTAAGGTCATCC | 52 | 152 |
|  | *tsp*E4.C2 -R | AGTTTATCGCTGCGGGTCGC | 63 |  |
| *arp*A | *arp*A -F | AACGCTATTCGCCAGCTTGC | 62 | 400 |
|  | *arp*A -R | TCTCCCCATACCGTACGCTA | 60 |  |

6 Primers for detection of Serotyping

| Gene | Prime name | Primer sequence （5’ – 3’） | Temperature of annealing（℃） | Length of PCR products (bp) |
| --- | --- | --- | --- | --- |
| *fli*C | H12-F | CAGCAAGCGGTGAAGTGAAC | 60 | 947 |
|  | H12-R | TGCAGCAGAGACAGAACCTG | 60 |  |
| *fli*C | H25 -F | GGGCTGAATGGCTTCTCAGT | 60 | 782 |
|  | H25 -R | TTGGACAGCACGGAGTTACC | 60 |  |
| *fliC* | H10 -F | CAGTAGGTGCTGGCGACTTT | 60 | 679 |
|  | H10 -R | TGCAGCAGAGACAGAACCTG | 60 |  |
| Wzt | O8 -F | GCGTCTACTCCAGTGGGATG | 59 | 649 |
|  | O8 -R | GCGACAGCGACAGAATAGGA | 59 |  |
| Wzx | O21-F | CGCTGTCAACGAGTGTGATG | 59 | 663 |
|  | O21-R | GGAGCAACAAACCGGAAAGT | 58 |  |
| Wzx | O40 -F | TTCATTGCAAAGGCTACGGT | 58 | 684 |
|  | O40 -R | ACCCCATACTATTGCCACCC | 59 |  |
| Wzx | O75 -F | AACCGCTCAGCGAGAATGAA | 60 | 880 |
|  | O75 -R | TCAGAAGGTACCCTGACAACAC | 59 |  |
| Wzt | O153-F | ACGTATTCTCATGCCAGTTCCT | 59 | 650 |
|  | O153-R | GCCTGAACACGGAAGATAAACC | 59 |  |
| *fli*C | H7-F | GAGCGTCTGTCTTCTGGCTT | 60 | 1573 |
|  | H7-R | TGGACACTTCGGTCGCATAG | 59 |  |
| *flk*A | H47 -F | GAGTTCCGCTATCGAGCGTC | 60 | 1006 |
|  | H47 -R | AGTTGTCTGGTTAGCCTGCG | 60 |  |
| Wzx | O87-F | GGTTCTTGGGCCGGAGTTTT | 60 | 1070 |
|  | O87-R | TTGCTATCGCAGCACCATCT | 59 |  |
| Wzx | O147 -F | GCAATTCCTGCGTTAGGTATACTG | 59 | 1113 |
|  | O147 -R | TGCTATTGCGGCTCCAACAA | 60 |  |
| Wzx | O185-F | TGCAAGAGCTCTGAGCCAAT | 59 | 1321 |
|  | O185-R | ACAAACATAACTCCCTGCACCT | 59 |  |

7 Primers for detection of ARGs

| Gene | Prime name | Primer sequence （5’ – 3’） | Temperature of annealing（℃） | Length of PCR products (bp) |
| --- | --- | --- | --- | --- |
| *aad*A | *aad*A1-F | GAGCGCCATCTCGAACCGAC | 62 | 496 |
|  | *aad*A1-R | CATTTCGCTCATCGCCAGCC | 61 |  |
| *aad*A | *aad*A5-F | TTTTCCCTGCACAAGTTTTCAAGC | 61 | 753 |
|  | *aad*A5-R | CTTTGGCATATCGAACGAACGC | 60 |  |
| *aad*A | *aad*A8b-F | TGAGCGCCATCTGGAATCAA | 59 | 526 |
|  | *aad*A8b-R | GTACCAAATGCGGGACAACG | 59 |  |
| APH | APH(3')-Ia-F | TCAACGGGAAACGTCTTGCT | 60 | 729 |
|  | APH(3')-Ia-R | AGCCGTTTCTGTAATGAAGGAGA | 59 |  |
| APH | APH(3'')-Ib -F | GTTTTTCGACGTGGTGACGG | 60 | 725 |
|  | APH(3'')-Ib-R | CAACCCCAAGTCAGAGGGTC | 59 |  |
| APH | APH(6)-Id -F | GCGTTGCTCCTCTTCTCCAT | 60 | 681 |
|  | APH(6)-Id -R | ACTCCTGCAATCGTCAAGGG | 60 |  |
| *bla ampC* | *bla* ampC -F | CCACATTTGCTGCCCCCCAAA | 63 | 781 |
|  | *bla* ampC -R | CAGTAGCGAGATTGTGCCAC | 58 |  |
| *bla*EC | *bla*EC -F | CCGCCTCTTGCTCCACATTTG | 60 | 793 |
|  | *bla*EC -R | CAGTAGCGAGATTGTGCCAG | 58 |  |
| *bla*TEM | *bla*TEM -F | CGGCATTTTGCCTTCCTGTT | 59 | 762 |
|  | *bla*TEM -R | CCATAGTTGCCTGACTCCCC | 59 |  |
| *dfr*A | *dfr*A1-F | GGAGTTATCGGGAATGGCCC | 60 | 336 |
|  | *dfr*A1-R | ACCTTCCGGCTCGATGTCTA | 60 |  |
| *dfr*A | *dfr*A12-F | AACGGAGTGGGTGTACGGAA | 61 | 460 |
|  | *dfr*A12-R | ACGCATTTATCTCGTTGCTGC | 59 |  |
| *dfr*A | *dfr*A14-F | GATGGCTGCGAAAGCGAAAA | 60 | 412 |
|  | *dfr*A14-R | GTGTTGCTCAAAAACAACTTCG | 57 |  |
| *dfr*A | *dfr*A17-F | CCAAATCTGGTATGTATAAT | 47 | 431 |
|  | *dfr*A17-R | AAAATGGCGTAATCGGTAGT | 55 |  |
| *qnrS* | QnrS1 -F | TCGGCACCACAACTTTTCAC | 59 | 559 |
|  | QnrS1 -R | TGTATCGACTTTGCGGGGAT | 59 |  |
| *flo*R | *flo*R -F | TGCTGATGGCTCCTTTCGAC | 60 | 1115 |
|  | *flo*R -R | CAGCCCCAACGAAACCAGTA | 60 |  |
| *sul* | *sul*2-F | ATGAAGTCAGCTCCACCTGC | 60 | 715 |
|  | *sul*2-R | TCGGCATCGTCAACATAACCTT | 59 |  |
| *sul* | *Sul*3-F | ACCGATAGTTTTTCCGATGGAGG | 60 | 716 |
|  | *Sul*3-R | TTAACATCATGGGTGCGGAGA | 59 |  |
| *tet*A | *tet*A -F | TGAAACCCAACAGACCCCTG | 59 | 1000 |
|  | *tet*A -R | CCCTGACGTTCCTCATCCAC | 60 |  |
