# Supplementary material for: Emergence of highly virulent and multidrug-resistant Escherichia coli in breeding sheep with pneumonia, Hainan Province, China
Source: Front Microbiol. 2024 Oct 23;15:1479759. doi: 10.3389/fmicb.2024.1479759 (PMC11539166; doi:10.3389/fmicb.2024.1479759)
Supplement: Supplementary file 2 [file Table_2.docx]

**Table S2: All strain information with the same genotype as this study in the pubMLST database**

| id | isolate | species | country | **region** | disease | source | detailed_source | phylogroup | ST (MLST (Achtman)) | ST (MLST (Pasteur)) |
| --- | --- | --- | --- | --- | --- | --- | --- | --- | --- | --- |
| 11812 | Y13F1 | Escherichia coli | China | Hainan province | pneumonia | animal | Sheep's lungs | B1 | 971 | 87 |
| 17808 | 562.60615 | Escherichia coli | China | China: Sichuan |  | animal | Pig | B1 | 971 | 87 |
| 19114 | 562.795 | Escherichia coli | China | China: Henan |  | animal | Sheep | B1 | 971 | 87 |
| 19160 | 562.79562 | Escherichia coli | China | China: Jiangsu |  | animal | Sheep | B1 | 971 |  |
| 19162 | 562.79565 | Escherichia coli | China | China: Jiangsu |  | animal | Sheep | B1 | 971 |  |
| 19163 | 562.79567 | Escherichia coli | China | China: Jiangsu |  | animal | Sheep | B1 | 971 |  |
| 19164 | 562.79569 | Escherichia coli | China | China: Jiangsu |  | animal | Sheep | B1 | 971 |  |
| 19165 | 562.7957 | Escherichia coli | China | China: Jiangsu |  | animal | Sheep | B1 | 971 |  |
| 19852 | A267 | Escherichia coli | China |  | healthy | human | Feces |  | 971 | 87 |
| 3116 | Nordic_E38 | Escherichia coli | Denmark |  |  | animal | diseased bird |  | 1146 |  |
| 11789 | Y2F2 | Escherichia coli | China | Hainan province | pneumonia | animal | Sheep's lungs | B1 | 1146 | 1106 |
| 11807 | Y2Q1 | Escherichia coli | China | Hainan province | pneumonia | animal | Sheep's trachea | B1 | 1146 | 1106 |
| 12578 | PDT000934779.1 | Escherichia coli | Kenya |  |  | animal | bovine |  | 1146 | 1106 |
| 14941 | 562.21433 | Escherichia coli | USA | USA: IA |  | animal | Cow | B1 | 1146 |  |
| 15169 | 562.31142 | Escherichia coli | UK | United Kingdom |  | animal | Cow | B1 | 1146 | 1106 |
| 4239 | 83-G22-A | Escherichia coli | France |  | healthy | animal | bovine commensal (face) |  | 1308 | 1314 |
| 4254 | 99-K118-A | Escherichia coli | France |  | healthy | animal | bovine commensal (face) |  | 1308 | 1314 |
| 11811 | Y11S2 | Escherichia coli | China | Hainan province | pneumonia | animal | Sheep's kidney | B1 | 1308 | 1114 |
| 12514 | PDT000935250.1 | Escherichia coli | Kenya |  |  | animal | chickens |  | 1308 | 1314 |
| 12581 | PDT000934762.1 | Escherichia coli | Kenya |  |  | animal | rodent |  | 1308 | 1314 |
| 14501 | 2056315.7 | Escherichia coli | South Africa | Mogosane, North-West Province |  | animal | Cow | B1 | 1308 | 1314 |
| 14808 | 562.18715 | Escherichia coli | USA | USA:CO |  | animal | Cow | B1 | 1308 |  |
| 15134 | 562.31069 | Escherichia coli | UK | United Kingdom |  | animal | Cow | B1 | 1308 |  |
| 15338 | 562.46059 | Escherichia coli | USA | USA: Alachua, FL |  | animal | Cow | B1 | 1308 | 1314 |
| 15340 | 562.46061 | Escherichia coli | USA | USA: Alachua, FL |  | animal | Cow | B1 | 1308 | 1314 |
| 15615 | 562.47262 | Escherichia coli | Japan |  |  | animal | Cow | B1 | 1308 | 1314 |
| 15776 | 562.47424 | Escherichia coli | Japan |  |  | animal | Cow | B1 | 1308 | 956 |
| 15809 | 562.47457 | Escherichia coli | Japan |  |  | animal | Cow | B1 | 1308 |  |
| 16010 | 562.47673 | Escherichia coli | France |  |  | animal | Cow | B1 | 1308 | 1314 |
| 16025 | 562.47688 | Escherichia coli | France |  |  | animal | Cow | B1 | 1308 | 1314 |
| 18483 | 562.62982 | Escherichia coli | Peru | Peru: Lima |  | animal | Chicken | B1 | 1308 |  |
| 19608 | 19ZY255 | Escherichia coli | China |  | healthy | human | Feces |  | 1308 | 1314 |
| 19689 | AH47 | Escherichia coli | China |  | healthy | human | Feces |  | 1308 |  |
| 19873 | A97 | Escherichia coli | China |  | healthy | human | Feces |  | 1308 | 1314 |
| 20235 | 005-005-c2 | Escherichia coli | France | Paris | healthy | human | Feces |  | 1308 | 1314 |
| 20745 | MVAST0103 | Escherichia coli | USA | Minneapolis | bacteraemia | clinical | urine |  | 1308 | 1314 |
| 20972 | E1657 | Escherichia coli | Indonesia |  | diarrhoea | human | Traveller/Soldier |  | 1308 | 956 |
| 11805 | Y2G3 | Escherichia coli | China | Hainan province | pneumonia | animal | Sheep's liver | B1 | 1704 | 1107 |
| 15089 | 562.28367 | Escherichia coli | China | China: Xinjiang |  | animal | Cow | B1 | 1704 |  |
| 4234 | 78-A86-A | Escherichia coli | France |  | healthy | animal | bovine commensal (face) |  | 2521 |  |
| 11791 | Y2S3 | Escherichia coli | China | Hainan province | pneumonia | animal | Sheep's kidney | B1 | 2521 | 1109 |
| 11810 | Y5YF3 | Escherichia coli | China | Hainan province | pneumonia | animal | Sheep's lungs | B1 | 2521 | 1110 |
| 12572 | PDT000934806.1 | Escherichia coli | Kenya |  |  | animal | rabbit |  | 2521 |  |
| 12637 | PDT000934330.1 | Escherichia coli | Kenya |  |  | animal | chickens |  | 2521 |  |
| 14964 | 562.21809 | Escherichia coli | USA | USA: MD |  | animal | Cow | B1 | 2521 |  |
| 14991 | 562.25155 | Escherichia coli | USA | USA: MI |  | animal | Cow | B1 | 2521 |  |
| 15222 | 562.31392 | Escherichia coli | UK | United Kingdom |  | animal | Cow | B1 | 2521 | 1110 |
| 16005 | 562.47668 | Escherichia coli | France |  |  | animal | Cow | B1 | 2521 |  |
| 17300 | 562.19477 | Escherichia coli | USA | USA:CO |  | animal | Pig | B1 | 2521 |  |
| 20645 | 308_PUTI_Fec | Escherichia coli | USA | Minneapolis |  | clinical | Feces |  | 2521 |  |
| 21237 | EM1_Wa21 | Escherichia coli | Italy |  |  | animal | turtledove | B1 | 2521 |  |
| 21252 | EM1_Ca19 | Escherichia coli | Italy |  |  | animal | companion animal (face) | B1 | 2521 | 1110 |
| 11806 | Y2P1 | Escherichia coli | China | Hainan province | pneumonia | animal | Sheep's spleen | D | 5748 | 1090 |
| 15146 | 562.31094 | Escherichia coli | UK | United Kingdom |  | animal | Cow | D | 5748 |  |
| 11808 | Y3X1 | Escherichia coli | China | Hainan province | pneumonia | animal | Sheep's heart | A | 6335 | 1111 |
| 11809 | Y3YF3 | Escherichia coli | China | Hainan province | pneumonia | animal | Sheep's lungs | A | 6335 | 1111 |
| 12774 | PDT000934918.1 | Escherichia coli | Kenya |  |  | human | unknow |  | 6335 | 1111 |
| 19755 | A29 | Escherichia coli | China |  | healthy | human | Feces |  | 6335 | 1111 |
| 11824 | Y2XX1 | Escherichia coli | China | Hainan province | pneumonia | animal | sheep | B1 | 7117 | 21 |
| 11790 | Y2P2 | Escherichia coli | China | Hainan province | pneumonia | animal | Sheep's spleen | B1 | 14878 | 1107 |

**# The yellow table represents the strain information of this study**
