# Supplementary figures and images for: Emergence of highly virulent and multidrug-resistant Escherichia coli in breeding sheep with pneumonia, Hainan Province, China
Source: Front Microbiol. 2024 Oct 23;15:1479759. doi: 10.3389/fmicb.2024.1479759 (PMC11539166; doi:10.3389/fmicb.2024.1479759)

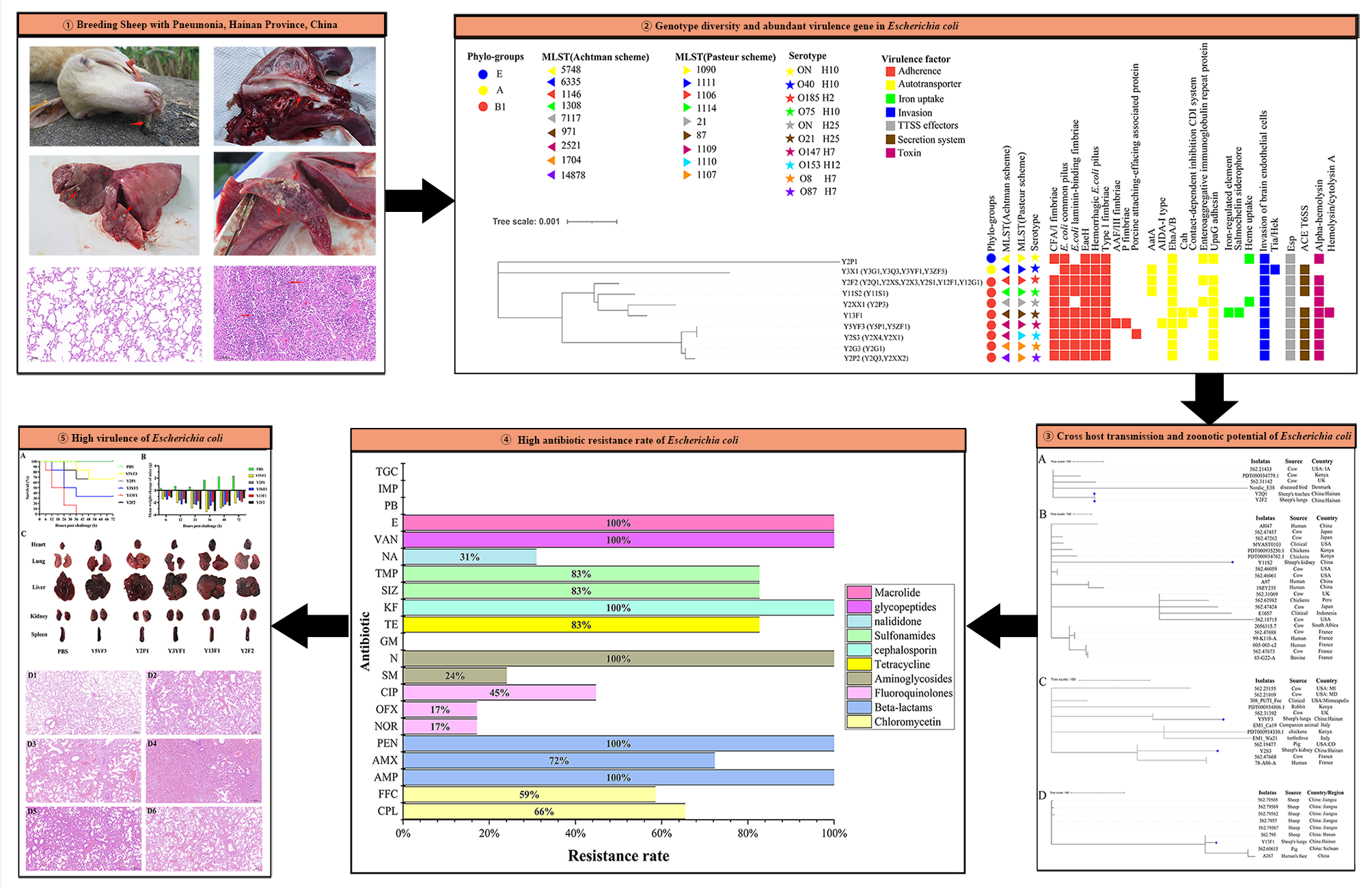

Supplement: Supplementary file 3 [file Image_1.TIF]
